# Synthesis and structure of a 1:1 co-crystal of hexa­methyl­ene­tetra­mine carb­oxy­borane and acetamino­phen

**DOI:** 10.1107/S2056989020015327

**Published:** 2020-11-24

**Authors:** Theppawut Ayudhya, Casey Raymond, Nin Dingra

**Affiliations:** a Department of Chemistry, University of Texas Permian Basin, Odessa, Texas, USA; b Department of Chemistry, State University of New York at Oswego, Oswego, New York, USA

**Keywords:** co-crystal structure, amine carb­oxy­borane, acetamino­phen, CORCB

## Abstract

Hexa­methyl­ene­tetra­mine carboacetamino­phenborane, a mol­ecule with two pharmacophores attached to a central carb­oxy­borate moiety, was synthesized and crystals were grown with an acetamino­phen co-crystal former to result in the title 1:1 co-crystal [hexa­methyl­ene­tetra­mine 4-acetamido­phenyl 2-boranyl­acetate–4-acet­amido­phenol (1/1)], C_15_H_22_BN_5_O_3_·C_8_H_9_NO_2_. In the first of these mol­ecules, both the borate-ester and acetyl­amino groups are considerably twisted away from the plane of the inter­vening benzene ring [dihedral angles = 76.89 (9) and 65.42 (9)°, respectively]. The extended structure of this co-crystal features N—H⋯O and O—H⋯O hydrogen bonds, which link the components into (100) sheets and weak C—H⋯O hydrogen bonds help to consolidate the structure.

## Abstract 2

Acetamino­phen assists as a co-former in the crystallization of hexa­methyl­ene­tetra­mine carboacetamino­phenborane, which may otherwise be challenging to form a crystal itself.

## Chemical context   

Crystal structures of pure drugs are of great inter­est in the pharmaceutical industry since these structures provide an understanding of the inter­molecular inter­actions that explain the physical and chemical properties of the solid (Desiraju, 2007 ▸). Modifications made to the active pharmaceutical ingredients to enhance the biological availability often include crystal engineering. For instance, the recrystallization of acetamino­phen, C_8_H_9_NO_2_ (also known as paracetamol), gives crystal form II, which displays better solubility and compressibility than form I (Naumov *et al.*, 1998 ▸; Agnew *et al.*, 2016 ▸). Another approach that has been brought into attention is using crystal formers or co-formers to improve the physicochemical characteristics of the solids. Recent developments in co-crystallization show potential advantages of drug–coformer co-crystals as well as drug–drug co-crystals (Kaur *et al.*, 2017 ▸; Cheney *et al.*, 2011 ▸; Nugrahani *et al.*, 2007 ▸; Dalpiaz *et al.*, 2018 ▸).

A group of organo–boron compounds, namely amine carb­oxy­boranes, have been studied extensively for their diverse biological effects such as anti-inflammatory, anti-neoplastic and anti-osteoporotic activities (Hall *et al.*, 1995 ▸, 1990 ▸; Murphy *et al.*, 1996 ▸). Their fundamental structure contains tetra­valent amines connected to a boron atom of the carb­oxy­borane moiety with an N—B coordinate covalent bond (Spielvogel *et al.*, 1976 ▸). As a result of the ease of structural transformability, this group is very amenable to modification such as exchanging various amine groups and esterification on the carb­oxy­borate. Our inter­est in amine carb­oxy­boranes stemmed from their innate structure that undergoes deca­rbonylation to produce CO, H_2_, and the amine group when placed in aqueous solution. We have shown amine carb­oxy­boranes to be a group of mol­ecules that can be used as carbon monoxide releasers (Ayudhya *et al.*, 2017 ▸). Moreover, we have recently reported that this process is accelerated by reactive oxygen species (ROS) increasing the rate at which CO and the amine group is released (Ayudhya *et al.*, 2018 ▸). Considering the amine compounds are drug mol­ecules, carb­oxy­boranes can be used as a system to deliver drugs that contain amino groups. Since we started our endeavor with drug-conjugated carb­oxy­boranes (Ayudhya *et al.*, 2018 ▸), we speculated that carb­oxy­boranes may be able to carry more than one drug. In addition to the amine group on the boron atom, ester and amide derivatives at the carb­oxy­borate end have been shown previously (Das *et al.*, 1990 ▸).
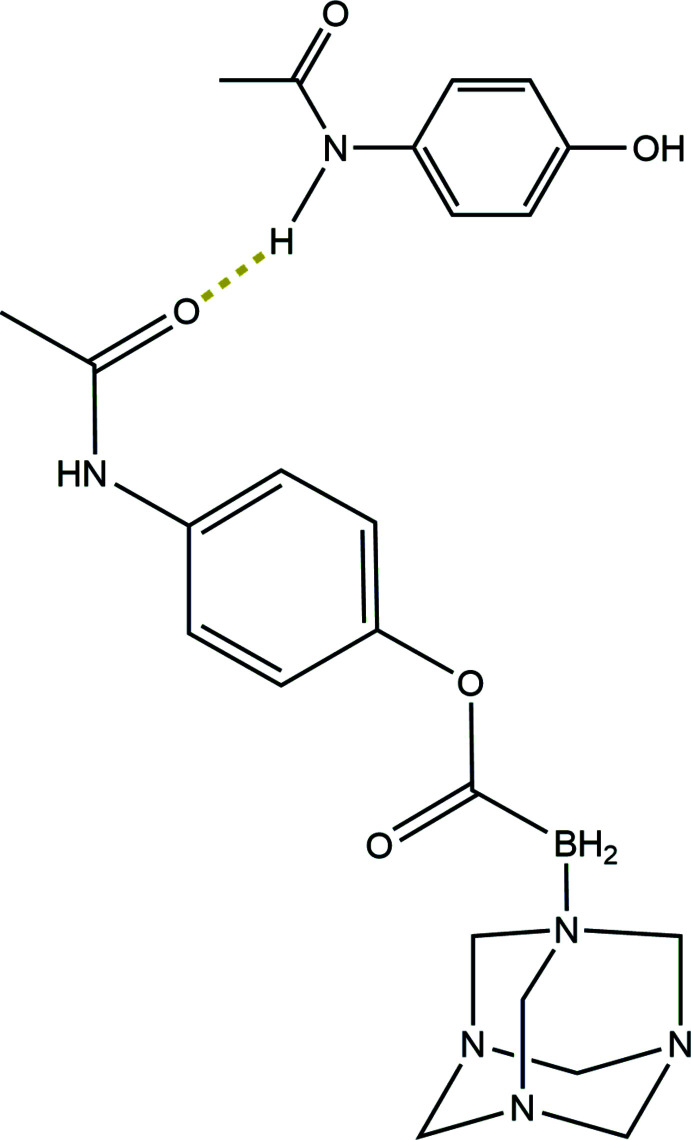


As part of this work, we now describe the crystal structure of the title co-crystal, C_15_H_22_BN_5_O_3_·C_8_H_9_NO_2_, (I), which resulted from the synthetic concept that conjugating two different pharmacophores to the carb­oxy­borate moiety may make a mol­ecule that has multiple biological effects.

## Structural commentary   

**Figure 1:**
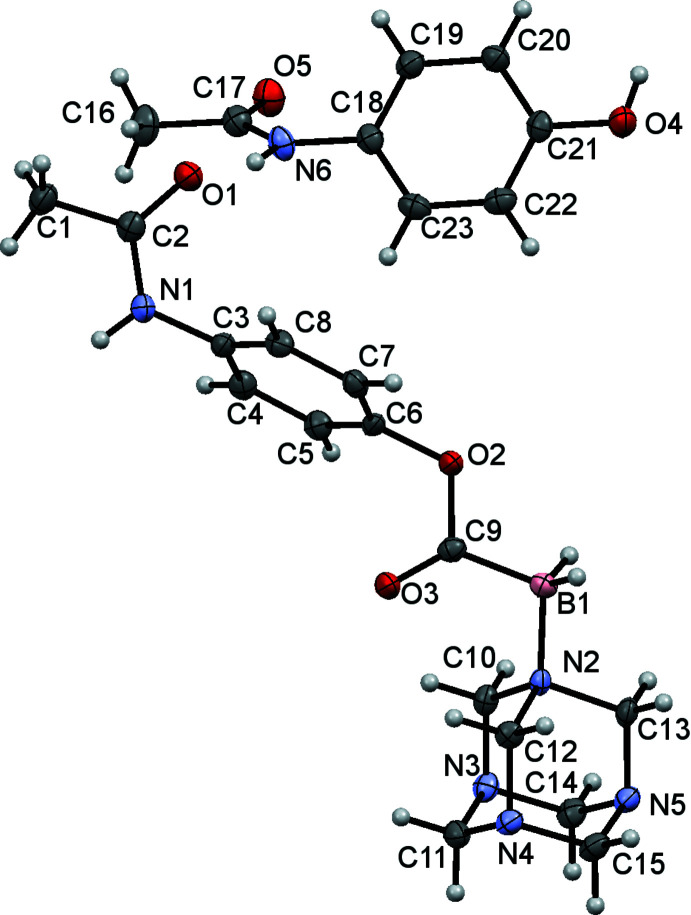
The mol­ecular structure of (I) with displacement ellipsoids drawn at the 50% probability level.

The asymmetric unit of the resulting monoclinic crystal (space group *P*2_1_/*c*) contains one C_15_H_22_BN_5_O_3_ ester (CORCB-1-APAP) and one C_8_H_9_NO_2_ acetamino­phen mol­ecule (Fig. 1 ▸). The hexa­methyl­ene­tetra­amine (hmta) moiety of the ester is *syn* to the C9=O3 carb­oxy carbonyl group and the aromatic C3–C8 ring is approximately perpendicular to the plane of the B1/C9/O2/O3 ester carboxyl­ate group [dihedral angle = 76.89 (9)°] while the C1/C2/N1/O1 acetyl­amino group is twisted out of plane of the ring by 65.42 (9)°; the dihedral angle between the pendant groups is 11.70 (10)°.

Based on the observed geometry, we may assume that the bonding in this difunctionalized carb­oxy­borate is very similar to that in the previously reported crystal structure of C_7_H_15_BN_4_O_2_ or CORCB-1 [Ayudhya *et al.*, 2017 ▸; Cambridge Structural Database (Groom *et al.*, 2016 ▸) refcode UDAQOI]. The only significant difference is in the slightly longer C9—O2 single bond, 1.399 (2) Å in the difunctionalized title compound compared to 1.353 (3) Å in CORCB-1. This lengthening is expected to be due to the weak ester bond, which is confirmed by rapid hydrolysis. There are only small differences in B—N and B—C bond lengths between the two materials with some lengthening seen in the difunctionalized compound. In the co-crystallized acetamino­phen mol­ecule in (I), the dihedral angle between the C18–C23 benzene ring and the acetyl­amino C16/C17/N6/O5 grouping is 54.61 (10)°.

## Supra­molecular features   

**Figure 2:**
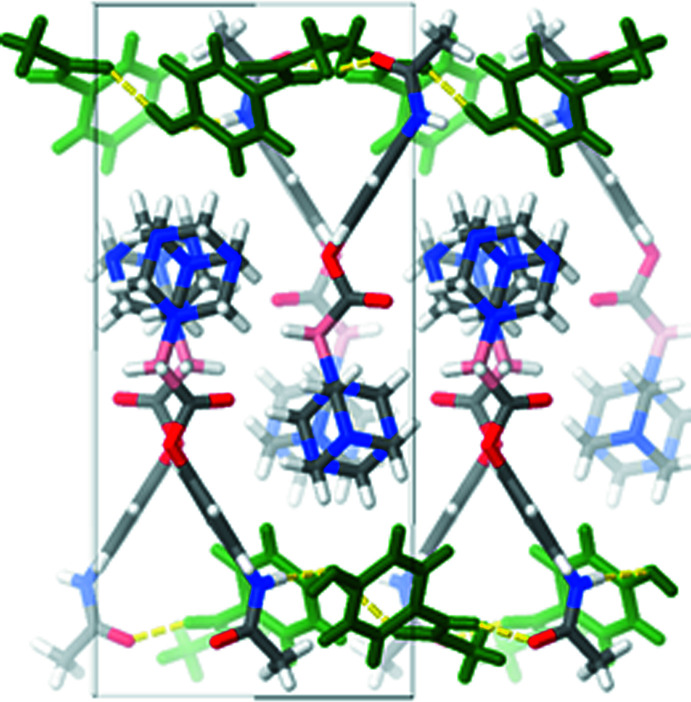
Unit cell packing of (I) viewed down the *c-*axis direction, with additional mol­ecules added along the *b-*axis direction. Acetamino­phen mol­ecules are green; hydrogen bonds are shown as dashed yellow lines.

During crystallization, the new difunctionalized mol­ecule, CORCB-1-APAP, forms a co-crystal with acetamino­phen at a 1:1 ratio with hydrogen-bonding inter­actions (Table 1 ▸) between them (Figs. 2 ▸ and 3 ▸). In comparison to the CORCB-1 crystal reported previously, which features hydrogen bonds between the amino and carb­oxy­lic acid groups (Ayudhya *et al.*, 2017 ▸), this new structure cannot form hydrogen bonds in the CORCB-1 region due to the replacement of carb­oxy­lic acid with an ester functional group. As a result, a co-crystal former such as acetamino­phen is needed for crystal formation to provide stable hydrogen bonds: with acetamino­phen mol­ecules flanking CORCB-1-APAP; no inter­actions are observed between these difunctionalized compounds. The co-crystal shows three classical hydrogen bonds. The first is an N6—H6*N*⋯O1 hydrogen bond (H⋯O = 2.00 Å) found between the N—H group of acetamino­phen and the C=O acceptor from CORCB-1-APAP. This type of bond has been previously reported in the acetamino­phen co-crystal with citric acid (Elbagerma *et al.*, 2011 ▸). Pure acetamino­phen crystals typically only form hydrogen bonds between N—H⋯O—H and O—H⋯O=C. The second inter­action N1—H1*N*⋯O4—H4*O* is between CORCB-1-APAP and another acetamino­phen mol­ecule. The bond length (2.18 Å) of this hydrogen bond is similar to the N—H⋯O—H bond (2.09 Å) from the known acetamino­phen crystal form II (Agnew *et al.*, 2016 ▸; Thomas *et al.*, 2011 ▸). The third hydrogen bond does not involve CORCB-1-APAP: it is exclusively formed between two acetamino­phen mol­ecules and this O4—H4*O*⋯O5=C17 bond (1.85 Å) is identical in length to that of acetamino­phen crystal form II (1.85 Å). Several weak C—H⋯O hydrogen bonds may help to consolidate the structure.

**Figure 4:**
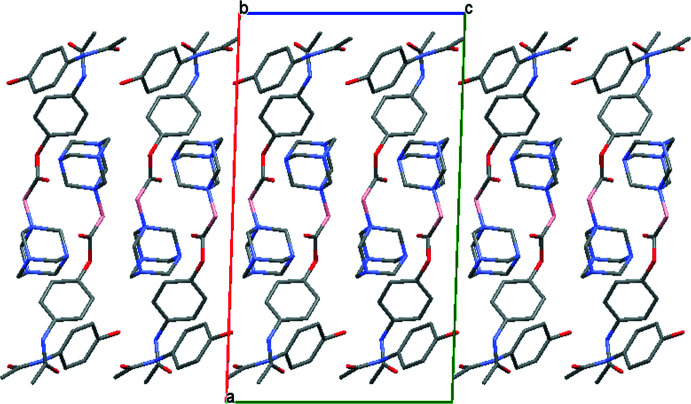
Mol­ecular packing diagram for (I) viewed down [010].

A mol­ecular packing projection of (I) is shown in Fig. 4 ▸ for clear representation of each pair of CORCB-1-APAP and its co-former, acetamino­phen. As noted, the observed hydrogen-bond lengths in this co-crystal are similar to those from acetamino­phen form II packing while the overall packing looks similar to form I (Naumov *et al.*, 1998 ▸).

## Database survey   

The crystal structures of amine carb­oxy­borane have been reported as dimers (Spielvogel *et al.*, 1980 ▸; Rana *et al.*, 2002 ▸; Vyakaranam *et al.*, 2002 ▸). The CORCB-1 crystal structure does not show typical hydrogen bonding from carb­oxy­lic acid groups and does not show dimer formation (Ayudhya *et al.*, 2017 ▸). Acetamino­phen co-crystallized structures to name a few are with ibuprofen (Stone *et al.*, 2009 ▸), citric acid (Elbagerma *et al.*, 2011 ▸), theophylline (Childs *et al.*, 2007 ▸) and morpholine (Oswald *et al.*, 2002 ▸).

## Synthesis and crystallization   

The synthesis of amine carb­oxy­borane derivatives such as methyl ester of various amine carb­oxy­borates have been described previously. Several esterification methods of amine carb­oxy­boranes with alcohols include using DCC to make 98% yield (Spielvogel *et al.*, 1986 ▸) and using a catalytic amount of hydrogen bromide, which provides nearly qu­anti­tative yields (Győri *et al.*, 1995 ▸). In our process, esterification is completed before the amine exchange reaction and not *vice versa*. The synthesis of hexa­methyl­ene­tetra­mine carbo­acet­amino­phenborane (CORCB-1-APAP) involves several steps using tri­methyl­amine carb­oxy­borane (CORCB-3) as the starting material. Tri­methyl­amine carb­oxy­borane, synthesized by the previously reported method (Spielvogel *et al.*, 1976 ▸) is first esterified at the carb­oxy­borate moiety with acetamino­phen (APAP). The esterification was carried out in a mixed solvent system of chloro­form and THF (1:1) at 313 to 318 K for five days and the crude product was purified by a series of recrystallizations. CORCB-1-APAP and acetamino­phen co-crystals for X-ray data collection were grown in mixed solvents of hexa­ne/chloro­form using the solution crystallization method.

## Refinement   

Crystal data, data collection and structure refinement details are summarized in Table 2 ▸. All hydrogen atoms were treated as riding atoms in geometrically idealized positions [N—H = 0.86, O—H = 0.82 and C—H = 0.93–0.97 Å with *U*_iso_(H) = 1.2*U*_eq_(N,O,C) or 1.2*U*_eq_(Cmeth­yl)].

## Supplementary Material

Crystal structure: contains datablock(s) I, global. DOI: 10.1107/S2056989020015327/hb7947sup1.cif

Structure factors: contains datablock(s) I. DOI: 10.1107/S2056989020015327/hb7947Isup2.hkl

CheckCIF. DOI: 10.1107/S2056989020015327/hb7947sup4.pdf

CCDC reference: 1828957

Additional supporting information:  crystallographic information; 3D view; checkCIF report

## Figures and Tables

**Figure 3:**
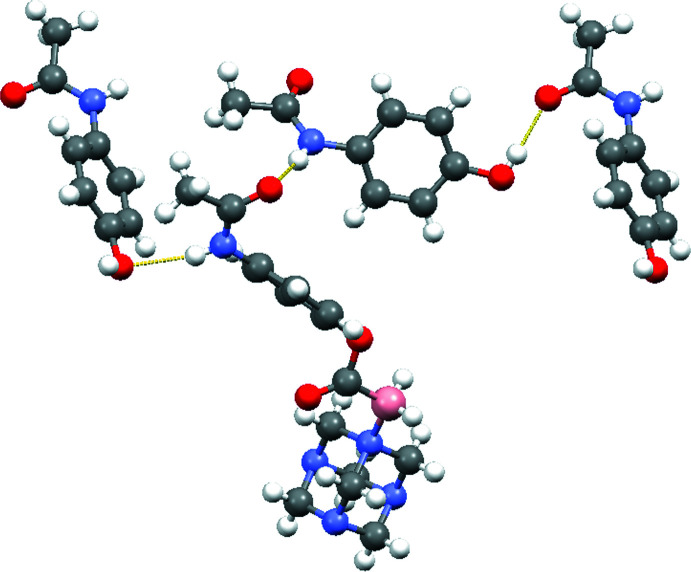
Detail of the packing of (I) showing hydrogen bonds (yellow lines) between the components of the co-crystal. Three acetamino­phen mol­ecules are shown but only the two on the left are hydrogen bonded with CORCB-1-APAP. The third acetamino­phen mol­ecule, which accepts a hydrogen bond from the second, is oriented in the same way as the first and repeats the pattern.

**Table 1:** Hydrogen-bond geometry (Å, °)

*D*—H⋯*A*	*D*—H	H⋯*A*	*D*⋯*A*	*D*—H⋯*A*
C10—H10*A*⋯O3	0.97	2.52	3.184 (2)	125
C12—H12*B*⋯O3	0.97	2.46	3.135 (2)	126
N1—H1*N*⋯O4^i^	0.86	2.18	3.0217 (19)	168
O4—H4*O*⋯O5^ii^	0.82	1.85	2.6619 (18)	174
N6—H6*N*⋯O1	0.86	2.00	2.8415 (19)	166
C10—H10*B*⋯O3^iii^	0.97	2.49	3.413 (2)	159
C15—H15*A*⋯O4^iv^	0.97	2.50	3.160 (2)	125
C20—H20⋯O5^ii^	0.93	2.51	3.195 (2)	130

Symmetry codes: (i) 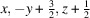; (ii) 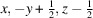; (iii) 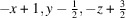; (iv) 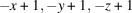.

**Table 2:** Experimental details

Crystal data	
Chemical formula	C_15_H_22_BN_5_O_3_·C_8_H_9_NO_2_
*M*_r_	482.35
Crystal system, space group	Monoclinic, *P*2_1_/*c*
Temperature (K)	293
*a*, *b*, *c* (Å)	20.760 (4), 9.5527 (19), 12.045 (2)
β (°)	91.929 (4)
*V* (Å^3^)	2387.2 (8)
*Z*	4
Radiation type	Mo *K*α
μ (mm^−1^)	0.10
Crystal size (mm)	0.23 × 0.06 × 0.05

Data collection	
Diffractometer	Bruker APEXII CCD
No. of measured, independent and observed [*I* > 2σ(*I*)] reflections	29824, 4870, 3044
*R*_int_	0.091
(sin θ/λ)_max_ (Å^−1^)	0.625

Refinement	
*R*[*F* ^2^ > 2σ(*F* ^2^)], *wR*(*F* ^2^), *S*	0.041, 0.072, 1.08
No. of reflections	4870
No. of parameters	319
H-atom treatment	H-atom parameters constrained
Δρ_max_, Δρ_min_ (e Å^−3^)	0.24, −0.22

Computer programs: *APEX2* and *SAINT* (Bruker, 2007 ▸), *SHELXS97* (Sheldrick, 2008 ▸), *SHELXL2014/7* (Sheldrick, 2015 ▸), *SHELXTL* (Sheldrick, 2008 ▸) and *publCIF* (Westrip, 2010 ▸).

## supplementary crystallographic information

### Crystal data

**Table:**

C_15_H_22_BN_5_O_3_·C_8_H_9_NO_2_	*F*(000) = 1024
*M**_r_* = 482.35	*D*_x_ = 1.342 Mg m^−^^3^
Monoclinic, *P*2_1_/*c*	Mo *K*α radiation, λ = 0.71073 Å
*a* = 20.760 (4) Å	Cell parameters from 7373 reflections
*b* = 9.5527 (19) Å	θ = 5.8–54.1°
*c* = 12.045 (2) Å	µ = 0.10 mm^−^^1^
β = 91.929 (4)°	*T* = 293 K
*V* = 2387.2 (8) Å^3^	Needle, colorless
*Z* = 4	0.23 × 0.06 × 0.05 mm

### Data collection

**Table:**

Bruker APEXII CCD diffractometer	*R*_int_ = 0.091
Radiation source: sealed tube	θ_max_ = 26.4°, θ_min_ = 2.0°
phi and ω scans	*h* = −25→25
29824 measured reflections	*k* = −11→11
4870 independent reflections	*l* = −15→15
3044 reflections with *I* > 2σ(*I*)	

### Refinement

**Table:**

Refinement on *F*^2^	Primary atom site location: structure-invariant direct methods
Least-squares matrix: full	Hydrogen site location: inferred from neighbouring sites
*R*[*F*^2^ > 2σ(*F*^2^)] = 0.041	H-atom parameters constrained
*wR*(*F*^2^) = 0.072	*w* = 1/[σ^2^(*F*_o_^2^) + (0.018*P*)^2^] where *P* = (*F*_o_^2^ + 2*F*_c_^2^)/3
*S* = 1.08	(Δ/σ)_max_ < 0.001
4870 reflections	Δρ_max_ = 0.24 e Å^−^^3^
319 parameters	Δρ_min_ = −0.22 e Å^−^^3^
0 restraints	

### Special details

**Table:**

Geometry. All esds (except the esd in the dihedral angle between two l.s. planes) are estimated using the full covariance matrix. The cell esds are taken into account individually in the estimation of esds in distances, angles and torsion angles; correlations between esds in cell parameters are only used when they are defined by crystal symmetry. An approximate (isotropic) treatment of cell esds is used for estimating esds involving l.s. planes.

### Fractional atomic coordinates and isotropic or equivalent isotropic displacement parameters (Å^2^)

**Table:**

	*x*	*y*	*z*	*U*_iso_*/*U*_eq_	
C1	0.05099 (9)	1.08726 (18)	0.86077 (16)	0.0285 (5)	
H1A	0.0175	1.1125	0.8076	0.043*	
H1B	0.0732	1.1701	0.8859	0.043*	
H1C	0.0324	1.0413	0.9229	0.043*	
C2	0.09783 (9)	0.98985 (19)	0.80733 (15)	0.0227 (4)	
O1	0.07872 (6)	0.88651 (12)	0.75419 (10)	0.0260 (3)	
N1	0.16095 (7)	1.02002 (14)	0.82219 (12)	0.0228 (4)	
H1N	0.1715	1.0898	0.8640	0.027*	
C3	0.21163 (8)	0.94201 (17)	0.77195 (15)	0.0194 (4)	
C4	0.25773 (9)	0.87339 (17)	0.83793 (16)	0.0221 (4)	
H4	0.2543	0.8727	0.9147	0.026*	
C5	0.30902 (9)	0.80567 (17)	0.78963 (15)	0.0217 (5)	
H5	0.3401	0.7601	0.8337	0.026*	
C6	0.31331 (8)	0.80687 (17)	0.67507 (16)	0.0186 (4)	
C7	0.26699 (8)	0.87268 (17)	0.60850 (15)	0.0209 (4)	
H7	0.2700	0.8718	0.5316	0.025*	
C8	0.21585 (9)	0.94021 (17)	0.65765 (15)	0.0214 (4)	
H8	0.1844	0.9843	0.6134	0.026*	
O2	0.36389 (6)	0.73601 (11)	0.62473 (10)	0.0221 (3)	
C9	0.42431 (8)	0.80105 (18)	0.63013 (15)	0.0189 (4)	
O3	0.42901 (6)	0.91608 (12)	0.67315 (10)	0.0242 (3)	
B1	0.47824 (10)	0.7044 (2)	0.57512 (19)	0.0210 (5)	
H1B1	0.4721	0.6078	0.5971	0.025*	
H1B2	0.4735	0.7095	0.4948	0.025*	
N2	0.54897 (7)	0.75441 (14)	0.61337 (11)	0.0164 (3)	
C10	0.55901 (8)	0.75507 (18)	0.73918 (14)	0.0191 (4)	
H10A	0.5288	0.8198	0.7712	0.023*	
H10B	0.5502	0.6624	0.7679	0.023*	
N3	0.62411 (7)	0.79543 (14)	0.77251 (12)	0.0206 (4)	
C11	0.63675 (9)	0.93610 (17)	0.72684 (15)	0.0236 (5)	
H11A	0.6065	1.0022	0.7571	0.028*	
H11B	0.6799	0.9653	0.7502	0.028*	
N4	0.63096 (7)	0.93989 (14)	0.60505 (12)	0.0197 (4)	
C12	0.56578 (8)	0.89959 (17)	0.57118 (15)	0.0191 (4)	
H12A	0.5615	0.9008	0.4907	0.023*	
H12B	0.5356	0.9672	0.5997	0.023*	
C13	0.59876 (8)	0.65314 (17)	0.56842 (15)	0.0209 (4)	
H13A	0.5903	0.5597	0.5958	0.025*	
H13B	0.5947	0.6509	0.4880	0.025*	
N5	0.66398 (7)	0.69366 (14)	0.60153 (12)	0.0202 (4)	
C14	0.66971 (9)	0.69583 (18)	0.72354 (15)	0.0234 (5)	
H14A	0.7134	0.7217	0.7462	0.028*	
H14B	0.6615	0.6026	0.7518	0.028*	
C15	0.67633 (9)	0.83683 (18)	0.56036 (16)	0.0244 (5)	
H15A	0.7201	0.8639	0.5816	0.029*	
H15B	0.6725	0.8372	0.4799	0.029*	
C16	0.06429 (10)	0.69737 (19)	0.99581 (16)	0.0331 (5)	
H16A	0.0271	0.6676	1.0348	0.050*	
H16B	0.0528	0.7742	0.9477	0.050*	
H16C	0.0974	0.7267	1.0484	0.050*	
C17	0.08889 (9)	0.57733 (19)	0.92747 (16)	0.0244 (5)	
O5	0.09247 (6)	0.45723 (12)	0.96712 (10)	0.0300 (3)	
N6	0.10633 (7)	0.60827 (14)	0.82433 (12)	0.0243 (4)	
H6N	0.1048	0.6947	0.8043	0.029*	
C18	0.12735 (9)	0.50769 (17)	0.74469 (15)	0.0211 (4)	
C19	0.08895 (9)	0.39203 (18)	0.71821 (15)	0.0227 (4)	
H19	0.0513	0.3762	0.7559	0.027*	
C20	0.10712 (9)	0.30031 (18)	0.63528 (15)	0.0232 (5)	
H20	0.0820	0.2221	0.6183	0.028*	
C21	0.16282 (9)	0.32553 (18)	0.57793 (15)	0.0213 (4)	
O4	0.17995 (6)	0.24556 (12)	0.48852 (11)	0.0272 (3)	
H4O	0.1529	0.1843	0.4771	0.033*	
C22	0.20183 (9)	0.43896 (18)	0.60524 (15)	0.0242 (5)	
H22	0.2398	0.4542	0.5681	0.029*	
C23	0.18357 (9)	0.53024 (18)	0.68920 (15)	0.0238 (5)	
H23	0.2095	0.6067	0.7078	0.029*	

### Atomic displacement parameters (Å^2^)

**Table:**

	*U*^11^	*U*^22^	*U*^33^	*U*^12^	*U*^13^	*U*^23^
C1	0.0260 (12)	0.0238 (10)	0.0363 (13)	0.0017 (9)	0.0109 (10)	−0.0002 (9)
C2	0.0258 (12)	0.0189 (10)	0.0237 (12)	0.0016 (9)	0.0041 (9)	0.0036 (9)
O1	0.0257 (8)	0.0202 (7)	0.0320 (8)	−0.0011 (6)	0.0027 (6)	−0.0018 (6)
N1	0.0211 (9)	0.0188 (8)	0.0286 (10)	0.0010 (7)	0.0035 (8)	−0.0056 (7)
C3	0.0181 (11)	0.0149 (10)	0.0254 (12)	−0.0012 (8)	0.0047 (9)	−0.0010 (8)
C4	0.0274 (12)	0.0188 (10)	0.0203 (11)	−0.0021 (9)	0.0053 (9)	0.0014 (8)
C5	0.0230 (11)	0.0159 (10)	0.0262 (12)	0.0018 (8)	0.0005 (9)	0.0032 (8)
C6	0.0155 (10)	0.0115 (9)	0.0292 (12)	−0.0014 (8)	0.0069 (9)	−0.0016 (8)
C7	0.0259 (11)	0.0175 (10)	0.0195 (11)	−0.0039 (8)	0.0032 (9)	−0.0016 (8)
C8	0.0201 (11)	0.0178 (10)	0.0260 (12)	0.0014 (8)	−0.0029 (9)	0.0015 (8)
O2	0.0177 (7)	0.0171 (7)	0.0316 (8)	−0.0019 (5)	0.0048 (6)	−0.0060 (6)
C9	0.0174 (10)	0.0182 (10)	0.0213 (11)	−0.0004 (8)	0.0017 (9)	0.0033 (8)
O3	0.0208 (7)	0.0172 (7)	0.0349 (8)	−0.0014 (6)	0.0043 (6)	−0.0062 (6)
B1	0.0214 (13)	0.0172 (11)	0.0246 (13)	−0.0034 (9)	0.0028 (10)	−0.0016 (9)
N2	0.0197 (9)	0.0131 (7)	0.0168 (9)	0.0019 (6)	0.0044 (7)	−0.0007 (6)
C10	0.0231 (11)	0.0170 (9)	0.0174 (11)	0.0035 (8)	0.0043 (9)	0.0020 (8)
N3	0.0205 (9)	0.0206 (8)	0.0209 (9)	0.0048 (7)	0.0004 (7)	0.0010 (7)
C11	0.0201 (11)	0.0193 (10)	0.0312 (12)	0.0006 (8)	−0.0001 (9)	−0.0042 (9)
N4	0.0167 (9)	0.0174 (8)	0.0252 (10)	0.0010 (7)	0.0034 (7)	0.0032 (7)
C12	0.0212 (11)	0.0157 (9)	0.0206 (11)	0.0002 (8)	0.0025 (9)	0.0053 (8)
C13	0.0221 (11)	0.0180 (10)	0.0231 (11)	0.0035 (8)	0.0071 (9)	−0.0027 (8)
N5	0.0171 (9)	0.0197 (8)	0.0239 (10)	0.0017 (7)	0.0047 (7)	0.0013 (7)
C14	0.0212 (11)	0.0215 (10)	0.0274 (12)	0.0041 (8)	0.0001 (9)	0.0030 (9)
C15	0.0192 (11)	0.0244 (11)	0.0298 (12)	0.0004 (8)	0.0061 (9)	0.0042 (9)
C16	0.0420 (14)	0.0275 (11)	0.0299 (13)	0.0095 (10)	0.0036 (11)	0.0009 (9)
C17	0.0259 (12)	0.0228 (11)	0.0245 (12)	0.0017 (9)	−0.0014 (10)	0.0003 (9)
O5	0.0389 (9)	0.0225 (7)	0.0287 (8)	0.0038 (6)	0.0033 (7)	0.0040 (6)
N6	0.0338 (10)	0.0145 (8)	0.0247 (10)	0.0014 (7)	0.0018 (8)	0.0004 (7)
C18	0.0241 (11)	0.0176 (10)	0.0216 (11)	0.0022 (9)	−0.0009 (9)	0.0010 (8)
C19	0.0205 (11)	0.0209 (10)	0.0270 (12)	0.0009 (8)	0.0037 (9)	0.0024 (9)
C20	0.0230 (11)	0.0171 (10)	0.0296 (12)	−0.0026 (8)	0.0035 (10)	0.0000 (9)
C21	0.0239 (11)	0.0166 (10)	0.0236 (12)	0.0047 (8)	0.0013 (9)	0.0018 (8)
O4	0.0275 (8)	0.0218 (7)	0.0326 (8)	−0.0007 (6)	0.0080 (7)	−0.0037 (6)
C22	0.0203 (11)	0.0249 (11)	0.0275 (12)	−0.0018 (9)	0.0013 (9)	0.0047 (9)
C23	0.0241 (12)	0.0199 (10)	0.0271 (12)	−0.0044 (8)	−0.0028 (10)	0.0020 (9)

### Geometric parameters (Å, º)

**Table:**

C1—C2	1.506 (2)	C11—H11B	0.9700
C1—H1A	0.9600	N4—C12	1.452 (2)
C1—H1B	0.9600	N4—C15	1.477 (2)
C1—H1C	0.9600	C12—H12A	0.9700
C2—O1	1.235 (2)	C12—H12B	0.9700
C2—N1	1.348 (2)	C13—N5	1.451 (2)
N1—C3	1.439 (2)	C13—H13A	0.9700
N1—H1N	0.8600	C13—H13B	0.9700
C3—C8	1.383 (2)	N5—C14	1.471 (2)
C3—C4	1.388 (2)	N5—C15	1.480 (2)
C4—C5	1.390 (2)	C14—H14A	0.9700
C4—H4	0.9300	C14—H14B	0.9700
C5—C6	1.386 (2)	C15—H15A	0.9700
C5—H5	0.9300	C15—H15B	0.9700
C6—C7	1.382 (2)	C16—C17	1.511 (2)
C6—O2	1.404 (2)	C16—H16A	0.9600
C7—C8	1.391 (2)	C16—H16B	0.9600
C7—H7	0.9300	C16—H16C	0.9600
C8—H8	0.9300	C17—O5	1.244 (2)
O2—C9	1.399 (2)	C17—N6	1.339 (2)
C9—O3	1.2174 (19)	N6—C18	1.436 (2)
C9—B1	1.611 (3)	N6—H6N	0.8600
B1—N2	1.597 (2)	C18—C23	1.381 (2)
B1—H1B1	0.9700	C18—C19	1.393 (2)
B1—H1B2	0.9700	C19—C20	1.390 (2)
N2—C10	1.523 (2)	C19—H19	0.9300
N2—C12	1.522 (2)	C20—C21	1.388 (2)
N2—C13	1.528 (2)	C20—H20	0.9300
C10—N3	1.449 (2)	C21—O4	1.377 (2)
C10—H10A	0.9700	C21—C22	1.386 (2)
C10—H10B	0.9700	O4—H4O	0.8200
N3—C11	1.479 (2)	C22—C23	1.397 (2)
N3—C14	1.479 (2)	C22—H22	0.9300
C11—N4	1.468 (2)	C23—H23	0.9300
C11—H11A	0.9700		
			
C2—C1—H1A	109.5	C12—N4—C11	108.57 (14)
C2—C1—H1B	109.5	C12—N4—C15	108.70 (14)
H1A—C1—H1B	109.5	C11—N4—C15	108.42 (14)
C2—C1—H1C	109.5	N4—C12—N2	111.71 (13)
H1A—C1—H1C	109.5	N4—C12—H12A	109.3
H1B—C1—H1C	109.5	N2—C12—H12A	109.3
O1—C2—N1	122.26 (17)	N4—C12—H12B	109.3
O1—C2—C1	120.98 (17)	N2—C12—H12B	109.3
N1—C2—C1	116.74 (16)	H12A—C12—H12B	107.9
C2—N1—C3	123.69 (15)	N5—C13—N2	111.70 (13)
C2—N1—H1N	118.2	N5—C13—H13A	109.3
C3—N1—H1N	118.2	N2—C13—H13A	109.3
C8—C3—C4	119.91 (17)	N5—C13—H13B	109.3
C8—C3—N1	119.80 (16)	N2—C13—H13B	109.3
C4—C3—N1	120.24 (16)	H13A—C13—H13B	107.9
C5—C4—C3	120.23 (17)	C13—N5—C14	108.77 (14)
C5—C4—H4	119.9	C13—N5—C15	108.97 (13)
C3—C4—H4	119.9	C14—N5—C15	108.22 (14)
C6—C5—C4	119.25 (17)	N5—C14—N3	112.10 (14)
C6—C5—H5	120.4	N5—C14—H14A	109.2
C4—C5—H5	120.4	N3—C14—H14A	109.2
C7—C6—C5	120.97 (17)	N5—C14—H14B	109.2
C7—C6—O2	118.98 (16)	N3—C14—H14B	109.2
C5—C6—O2	120.00 (16)	H14A—C14—H14B	107.9
C6—C7—C8	119.33 (17)	N4—C15—N5	111.96 (14)
C6—C7—H7	120.3	N4—C15—H15A	109.2
C8—C7—H7	120.3	N5—C15—H15A	109.2
C3—C8—C7	120.29 (17)	N4—C15—H15B	109.2
C3—C8—H8	119.9	N5—C15—H15B	109.2
C7—C8—H8	119.9	H15A—C15—H15B	107.9
C9—O2—C6	116.62 (13)	C17—C16—H16A	109.5
O3—C9—O2	118.65 (16)	C17—C16—H16B	109.5
O3—C9—B1	130.19 (16)	H16A—C16—H16B	109.5
O2—C9—B1	111.15 (14)	C17—C16—H16C	109.5
N2—B1—C9	110.79 (14)	H16A—C16—H16C	109.5
N2—B1—H1B1	109.5	H16B—C16—H16C	109.5
C9—B1—H1B1	109.5	O5—C17—N6	123.05 (17)
N2—B1—H1B2	109.5	O5—C17—C16	120.52 (17)
C9—B1—H1B2	109.5	N6—C17—C16	116.43 (16)
H1B1—B1—H1B2	108.1	C17—N6—C18	124.75 (15)
C10—N2—C12	107.66 (13)	C17—N6—H6N	117.6
C10—N2—C13	106.47 (13)	C18—N6—H6N	117.6
C12—N2—C13	107.04 (13)	C23—C18—C19	119.95 (17)
C10—N2—B1	112.47 (14)	C23—C18—N6	119.97 (16)
C12—N2—B1	113.27 (13)	C19—C18—N6	119.95 (16)
C13—N2—B1	109.56 (13)	C20—C19—C18	119.82 (18)
N3—C10—N2	111.84 (14)	C20—C19—H19	120.1
N3—C10—H10A	109.2	C18—C19—H19	120.1
N2—C10—H10A	109.2	C21—C20—C19	119.93 (17)
N3—C10—H10B	109.2	C21—C20—H20	120.0
N2—C10—H10B	109.2	C19—C20—H20	120.0
H10A—C10—H10B	107.9	O4—C21—C20	122.30 (16)
C10—N3—C11	108.32 (13)	O4—C21—C22	117.09 (16)
C10—N3—C14	108.77 (14)	C20—C21—C22	120.50 (17)
C11—N3—C14	108.20 (14)	C21—O4—H4O	109.5
N4—C11—N3	112.61 (14)	C21—C22—C23	119.29 (17)
N4—C11—H11A	109.1	C21—C22—H22	120.4
N3—C11—H11A	109.1	C23—C22—H22	120.4
N4—C11—H11B	109.1	C18—C23—C22	120.47 (17)
N3—C11—H11B	109.1	C18—C23—H23	119.8
H11A—C11—H11B	107.8	C22—C23—H23	119.8
			
O1—C2—N1—C3	4.5 (3)	C11—N4—C12—N2	−58.36 (17)
C1—C2—N1—C3	−176.59 (16)	C15—N4—C12—N2	59.38 (18)
C2—N1—C3—C8	64.0 (2)	C10—N2—C12—N4	56.54 (17)
C2—N1—C3—C4	−118.63 (19)	C13—N2—C12—N4	−57.59 (18)
C8—C3—C4—C5	1.6 (3)	B1—N2—C12—N4	−178.46 (14)
N1—C3—C4—C5	−175.82 (15)	C10—N2—C13—N5	−57.80 (17)
C3—C4—C5—C6	−0.4 (3)	C12—N2—C13—N5	57.13 (18)
C4—C5—C6—C7	−0.9 (3)	B1—N2—C13—N5	−179.67 (14)
C4—C5—C6—O2	−178.39 (15)	N2—C13—N5—C14	59.31 (18)
C5—C6—C7—C8	0.9 (2)	N2—C13—N5—C15	−58.47 (18)
O2—C6—C7—C8	178.43 (15)	C13—N5—C14—N3	−59.61 (18)
C4—C3—C8—C7	−1.5 (3)	C15—N5—C14—N3	58.64 (18)
N1—C3—C8—C7	175.85 (15)	C10—N3—C14—N5	59.51 (19)
C6—C7—C8—C3	0.3 (3)	C11—N3—C14—N5	−57.96 (18)
C7—C6—O2—C9	105.80 (17)	C12—N4—C15—N5	−59.85 (19)
C5—C6—O2—C9	−76.6 (2)	C11—N4—C15—N5	57.99 (18)
C6—O2—C9—O3	−3.1 (2)	C13—N5—C15—N4	59.57 (19)
C6—O2—C9—B1	176.90 (15)	C14—N5—C15—N4	−58.56 (18)
O3—C9—B1—N2	17.3 (3)	O5—C17—N6—C18	4.0 (3)
O2—C9—B1—N2	−162.61 (14)	C16—C17—N6—C18	−176.28 (16)
C9—B1—N2—C10	56.76 (19)	C17—N6—C18—C23	−130.07 (19)
C9—B1—N2—C12	−65.61 (19)	C17—N6—C18—C19	54.1 (2)
C9—B1—N2—C13	174.96 (14)	C23—C18—C19—C20	−0.5 (3)
C12—N2—C10—N3	−56.73 (17)	N6—C18—C19—C20	175.33 (16)
C13—N2—C10—N3	57.78 (17)	C18—C19—C20—C21	−1.1 (3)
B1—N2—C10—N3	177.79 (13)	C19—C20—C21—O4	−173.96 (16)
N2—C10—N3—C11	58.28 (17)	C19—C20—C21—C22	2.3 (3)
N2—C10—N3—C14	−59.11 (17)	O4—C21—C22—C23	174.61 (15)
C10—N3—C11—N4	−60.21 (18)	C20—C21—C22—C23	−1.8 (3)
C14—N3—C11—N4	57.55 (18)	C19—C18—C23—C22	1.0 (3)
N3—C11—N4—C12	60.24 (18)	N6—C18—C23—C22	−174.86 (16)
N3—C11—N4—C15	−57.68 (18)	C21—C22—C23—C18	0.2 (3)

### Hydrogen-bond geometry (Å, º)

**Table:**

*D*—H···*A*	*D*—H	H···*A*	*D*···*A*	*D*—H···*A*
C10—H10*A*···O3	0.97	2.52	3.184 (2)	125
C12—H12*B*···O3	0.97	2.46	3.135 (2)	126
N1—H1*N*···O4^i^	0.86	2.18	3.0217 (19)	168
O4—H4*O*···O5^ii^	0.82	1.85	2.6619 (18)	174
N6—H6*N*···O1	0.86	2.00	2.8415 (19)	166
C10—H10*B*···O3^iii^	0.97	2.49	3.413 (2)	159
C15—H15*A*···O4^iv^	0.97	2.50	3.160 (2)	125
C20—H20···O5^ii^	0.93	2.51	3.195 (2)	130

Symmetry codes: (i) *x*, −*y*+3/2, *z*+1/2; (ii) *x*, −*y*+1/2, *z*−1/2; (iii) −*x*+1, *y*−1/2, −*z*+3/2; (iv) −*x*+1, −*y*+1, −*z*+1.
