# Supplementary material for: Synthesis and structure of a 1:1 co-crystal of hexa­methyl­ene­tetra­mine carb­oxy­borane and acetamino­phen
Source: Acta Crystallogr E Crystallogr Commun. 2020 Nov 24;76(Pt 12):1854–8. doi: 10.1107/S2056989020015327 (PMC7784649; doi:10.1107/S2056989020015327)

checkCIF/PLATON report

Structure factors have been supplied for datablock(s) I

THIS REPORT IS FOR GUIDANCE ONLY. IF USED AS PART OF A REVIEW PROCEDURE FOR PUBLICATION, IT SHOULD NOT REPLACE THE EXPERTISE OF AN EXPERIENCED CRYSTALLOGRAPHIC REFEREE.

No syntax errors found. CIF dictionary Interpreting this report

Datablock: I

Bond precision: C-C = 0.0028 A Wavelength=0.71073

Cell:	a=20.760(4)	b=9.5527(19)	c=12.045(2)
	alpha=90	beta=91.929(4)	gamma=90
Temperature:	293 K		

	Calculated	Reported
Volume	2387.3(8)	2387.2(8)
Space group	P 21/c	P 21/c
Hall group	-P 2ybc	-P 2ybc
Moiety formula	C15 H22 B N5 O3, C8 H9 N O2	?
Sum formula	C23 H31 B N6 O5	C23 H31 B N6 O5
Mr	482.35	482.35
Dx,g cm-3	1.342	1.342
Z	4	4
Mu (mm-1)	0.096	0.096
F000	1024.0	1024.0
F000’	1024.44	
h,k,lmax	25,11,15	25,11,15
Nref	4870	4870
Tmin,Tmax	0.993,0.995	
Tmin’	0.978	

Correction method= Not given

Data completeness= 1.000 Theta(max)= 26.372

R(reflections)= 0.0413(3044) wR2(reflections)= 0.0720(4870)

S = 1.077 Npar= 319

The following ALERTS were generated. Each ALERT has the format
test-name_ALERT_alert-type_alert-level.
Click on the hyperlinks for more details of the test.

Alert level C

```
CRYSC01_ALERT_1_C The word below has not been recognised as a standard
            identifier.
            clear,
PLAT048_ALERT_1_C MoietyFormula Not Given (or Incomplete) ........     Please Check
PLAT906_ALERT_3_C Large K Value in the Analysis of Variance ......      2.177 Check
```

Alert level G

```
PLAT007_ALERT_5_G Number of Unrefined Donor-H Atoms .............          3 Report
PLAT199_ALERT_1_G Reported _cell_measurement_temperature ..... (K)       293 Check
PLAT200_ALERT_1_G Reported   _diffrn_ambient_temperature ..... (K)       293 Check
PLAT720_ALERT_4_G Number of Unusual/Non-Standard Labels ..........         2 Note
PLAT910_ALERT_3_G Missing # of FCF Reflection(s) Below Theta(Min).         1 Note
PLAT978_ALERT_2_G Number C-C Bonds with Positive Residual Density.         1 Info
```

```
   0 ALERT level A = Most likely a serious problem - resolve or explain
   0 ALERT level B = A potentially serious problem, consider carefully
   3 ALERT level C = Check. Ensure it is not caused by an omission or oversight
   6 ALERT level G = General information/check it is not something unexpected

   4 ALERT type 1 CIF construction/syntax error, inconsistent or missing data
   1 ALERT type 2 Indicator that the structure model may be wrong or deficient
   2 ALERT type 3 Indicator that the structure quality may be low
   1 ALERT type 4 Improvement, methodology, query or suggestion
   1 ALERT type 5 Informative message, check
```

Publication of your CIF

You should attempt to resolve as many as possible of the alerts in all categories. Often the minor alerts point to easily fixed oversights, errors and omissions in your CIF or refinement strategy, so attention to these fine details can be worthwhile. In order to resolve some of the more serious problems it may be necessary to carry out additional measurements or structure refinements. However, the nature of your study may justify the reported deviations from journal submission requirements and the more serious of these should be commented upon in the discussion or experimental section of a paper or in the "special_details" fields of the CIF. *checkCIF* was carefully designed to identify outliers and unusual parameters, but every test has its limitations and alerts that are not important in a particular case may appear. Conversely, the absence of alerts does not guarantee there are no aspects of the results needing attention. It is up to the individual to critically assess their own results and, if necessary, seek expert advice.

If you wish to submit your CIF for publication in Acta Crystallographica Section C or E, you should upload your CIF via the web. If you wish to submit your CIF for publication in IUCrData you should upload your CIF via the web. If your CIF is to form part of a submission to another IUCr journal, you will be asked, either during electronic submission or by the Co-editor handling your paper, to upload your CIF via our web site.

PLATON version of 19/10/2018; check.def file version of 15/10/2018

Datablock I - ellipsoid plot

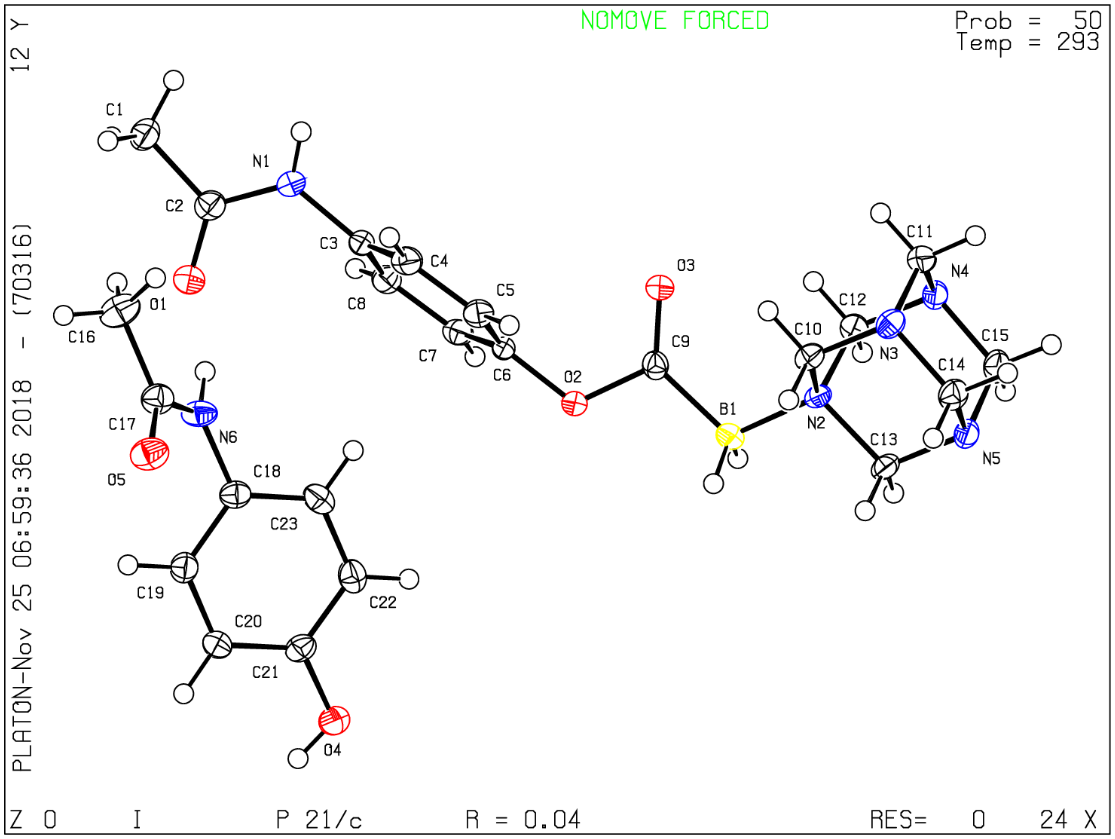

Supplement: Supplementary file 3 [file e-76-01854-sup4.pdf]
